# InterMine: a flexible data warehouse system for the integration and analysis of heterogeneous biological data

**DOI:** 10.1093/bioinformatics/bts577

**Published:** 2012-09-27

**Authors:** Richard N. Smith, Jelena Aleksic, Daniela Butano, Adrian Carr, Sergio Contrino, Fengyuan Hu, Mike Lyne, Rachel Lyne, Alex Kalderimis, Kim Rutherford, Radek Stepan, Julie Sullivan, Matthew Wakeling, Xavier Watkins, Gos Micklem

**Affiliations:** ^1^Department of Genetics, University of Cambridge, Cambridge CB2 3EH and ^2^Cambridge Systems Biology Centre, University of Cambridge, Cambridge CB2 1QR, UK

## Abstract

**Summary:** InterMine is an open-source data warehouse system that facilitates the building of databases with complex data integration requirements and a need for a fast customizable query facility. Using InterMine, large biological databases can be created from a range of heterogeneous data sources, and the extensible data model allows for easy integration of new data types. The analysis tools include a flexible query builder, genomic region search and a library of ‘widgets’ performing various statistical analyses. The results can be exported in many commonly used formats. InterMine is a fully extensible framework where developers can add new tools and functionality. Additionally, there is a comprehensive set of web services, for which client libraries are provided in five commonly used programming languages.

**Availability:** Freely available from http://www.intermine.org under the LGPL license.

**Contact:** g.micklem@gen.cam.ac.uk

**Supplementary information:** Supplementary data are available at *Bioinformatics* online.

## 1 INTRODUCTION

Integrative analysis exploiting diverse datasets is a powerful approach in modern biology, but it can also be complex and time consuming. There are huge quantities of data available in a range of different formats, and a high level of computational expertise is often required for performing analysis, even once the data have been successfully integrated into a single database [for a general review of biological data integration, see Triplet and Butler (2011)]. InterMine is a data warehouse framework initially developed for FlyMine to address these issues for the *Drosophila* community (Lyne *et al.*, 2007). It is being adopted by a number of major model organism databases including budding yeast, rat, zebrafish, mouse and nematode worm (Balakrishnan *et al.*, 2012; Shimoyama *et al.*, 2011), and is in use by the modENCODE project—a large-scale international initiative to characterize functional elements in the fly and worm genomes (Celniker *et al.*, 2009; Contrino *et al.*, 2012), as well as for drug target discovery (Chen *et al.*, 2011), *Drosophila* transcription factors (Pfreundt *et al.*, 2010) and mitochondrial proteomics (Smith *et al.*, 2012). Many features of the InterMine system have been designed to lower the effort required for the setup and maintenance of large-scale biological databases, and these are outlined later in the text and discussed in more detail in the Supplementary Material.

### 1.1 Data loading and integration

The InterMine database build system allows for the integration of datasets of varying size and complexity. It comes equipped with data integration modules that load data from common biological formats (e.g. GFF3, FASTA, OBO, BioPAX, GAF, PSI and chado—see Supplementary Material for a full list), while custom sources can be added by writing data parsers using the Java application programming interface (API) or by creating XML files in the appropriate format, for which a Perl API exists. InterMine’s core biological model is based on the Sequence Ontology (Eilbeck *et al.*, 2005) and can be easily extended by editing an XML file. All model-based components of the system are automatically derived from the data model, allowing simple error-free upgrading of the user interface and API as new data types are added.

One of the challenges of integrating data from different sources is that valuable datasets can become outdated when the gene models, and therefore identifiers, are updated. InterMine addresses this with an identifier resolver system that automatically replaces outdated identifiers with current ones when loading the dataset into the InterMine database. This is achieved using a map of current identifiers and synonyms from relevant databases—the identifiers matching a single synonym are changed to the current version, while the ones matching none or multiple synonyms are discarded. Integrating datasets can also be problematic if data values from different data sources conflict. To address this, database developers are able to set a priority for each data type, giving precedence to the more reliable data source at any level of data model granularity. To track data provenance, the database records each time a database row is created or modified.

### 1.2 Performance-tuned architecture

**Fig. 1.**
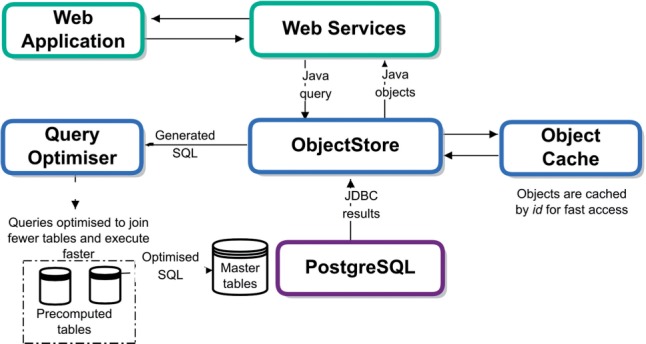
InterMine system architecture

At the core of InterMine is the ObjectStore, a custom object/relational mapping system written in Java and optimized for read-only database performance. The ObjectStore accepts object queries from a client (the web application or web services), generates SQL to execute in the underlying PostgreSQL database and materializes objects from the results (Fig. 1).

The Query optimizer improves performance using PostgreSQL tools and using pre-computed tables of results, which are created after all datasets have been loaded, joining connected data from different tables in the database. In this way, InterMine does not require, as other biological data warehouses do, a pre-defined database schema to perform. The analysis of estimated query execution times enables performance tuning, and query results are cached using a smart caching system, which can use previously run queries or parts of them to improve retrieval speed. This allows fast data mining of even large databases: modMine, the InterMine for the modENCODE project (Contrino *et al.*, 2012), currently contains >325 GB of data.

### 1.3 Data access

Users can access data both through RESTful web services and through the web application. The web application includes a faceted keyword search and powerful query tools. Users can input their own lists of identifiers (e.g. from genes), and lists and queries can be saved in a user’s private ‘MyMine’ account. Data identifiers are automatically updated to the most current ones for user input data, taking out a time-consuming step necessary for analysis across datasets. Type-specific report pages can be set up to provide collated summary information and relevant embedded interactive graphical displays (such as an interaction viewer, genome browser and expression heat maps), both for specific items such as individual genes and for lists of data items. The report pages provide summaries of results from a wide range of data types, facilitating serendipitous discovery.

InterMine provides a set of analysis ‘widgets’. These include tables (e.g. displaying orthologues and interactions), visual charts (e.g. displaying chromosome distribution graphs, gene expression results and interaction networks) and enrichment analysis widgets—see Supplementary Material for details (covering functional terms, protein domains and publications). Through a well-defined framework, developers can also create further widgets to customize the web application to their needs. Query ‘templates’ can easily be created for commonly run queries, and the QueryBuilder allows construction of advanced custom queries. InterMine also includes functionality for querying features with overlapping genome coordinates.

Query results can be saved and exported from the InterMine web application, e.g. as tab-delimited, comma-delimited, GFF3, FASTA or BED files, and can also be exported to Galaxy (Goecks *et al.*, 2010), where other data analysis workflows can be established and performed, such as analysing high-throughput sequencing data.

Web service APIs can be used to access the core functionality of InterMine such as uploading lists of data identifiers and running queries. They can also be used to access the metadata and specialized resources, such as retrieving enrichment statistics, running the region search or exploring the data model. Custom-written client libraries exist in a number of programming languages, including Python, Perl, Ruby, Java and JavaScript, enabling users to automate data-based workflows or access data directly without using the InterMine web application. The InterMine web application automatically generates code from queries for each of these client languages. In addition, query results are available in many formats including JSON or can be embedded directly in HTML, making it possible to view data from an InterMine database within another website (e.g. www.modencode.org). A recent use example of these facilities is the development of the YeastMine iPhone application (M. Cherry, personal communication).

## 2 CONCLUSIONS

Through its speed and flexibility, InterMine provides an advanced system for setting up biological data warehouses that facilitate bioinformatics analysis. As new data types emerge, the data model can be easily adapted to accommodate them. There is already a range of user-friendly tools, and the extensible framework means that specific analysis tools can be developed to cater to the changing needs of the users, making it particularly well-suited to the needs of biomedical researchers. Many of the major model organism databases are adopting InterMine as their data warehouse of choice, putting it in a good position to work on cross-organism interoperability as part of future development. There is an active InterMine developer community, and funding is in place until 2018, promising long-term sustainability of InterMine as a resource and continued improvements to meet researchers’ needs.

## Supplementary Material

Supplementary Data
