# Supplementary material for: InterMine: a flexible data warehouse system for the integration and analysis of heterogeneous biological data
Source: Bioinformatics. 2012 Sep 27;28(23):3163–5. doi: 10.1093/bioinformatics/bts577 (PMC3516146; doi:10.1093/bioinformatics/bts577)
Supplement: Supplementary Data [file supp_28_23_3163__index.html]

InterMine: a flexible data warehouse system for the integration and analysis of heterogeneous biological data — Supplementary Data 

# InterMine: a flexible data warehouse system for the integration and analysis of heterogeneous biological data

## Supplementary Data

files

**Files in this Data Supplement:**

- Supplementary Data - pdf file
