# Supplementary material for: InterMine: a flexible data warehouse system for the integration and analysis of heterogeneous biological data
Source: Bioinformatics. 2012 Sep 27;28(23):3163–5. doi: 10.1093/bioinformatics/bts577 (PMC3516146; doi:10.1093/bioinformatics/bts577)
Supplement: Supplementary Data [file supp_bts577_intermine_supplementary_materials.pdf]

InterMine: a flexible data warehouse system

Supplementary materials

Contents

1 Introduction **2**

2 About InterMine **2**

3 Data import and integration **3**
- 3.1 The data model . . . 3
- 3.2 Data import . . . 3
- 3.3 Extending the data model . . . 5
- 3.4 Data integration and quality checking . . . 5
- 3.5 Data types . . . 6

4 System architecture **6**
- 4.1 Object/Relational mapping . . . 6
- 4.2 Query Optimisation . . . 6

5 Web application **8**
- 5.1 Features . . . 8
- 5.2 User authentication and workspace . . . 9
- 5.3 Query capabilities . . . 12
- 5.4 Embedding external tools . . . 12

6 Web services **12**

7 Pros and cons - why use InterMine **14**
- 7.1 Comparison to existing systems . . . 15

1 Introduction

This document provides further details of how InterMine is implemented internally, as well as information on data types contained in different InterMine instances, statistics such as size and build time, and highlights of major features. It is intended as a more detailed guide to the system to anyone wishing to set up an InterMine database, as well as providing some background information to interested users. We also provide a discussion of the advantages and disadvantages of the InterMine system, in order to help potential users decide whether InterMine is a suitable application for their intended use.

2 About InterMine

InterMine is a data warehouse system suitable for any size of database (including very large ones), designed specifically to enable the integration of datasets of varying size, quality and complexity. It comes with features such as optimised query facilities, a web application with analysis and visualisation tools, and web services with libraries available in five languages. InterMine is an open source software project and is freely available under the LGPL license. More information about the project and links to the source code can be found on the project website at http://www.intermine.org, with a guide to setting up an InterMine database provided at http://www.intermine.org/wiki/GettingStarted.

	**Build time**	**RAM (build machine)**	**Number of objects**	**Database size without precomputes**	**Database size with precomputes**
**FlyMine**	14 hours	96 GB	29,276,415	75 GB	167 GB
**metabolicMine**	48 hours	96 GB	264,729,080	484 GB	1994 GB
**modMine**	3 days	24 GB	130,000,000	325 GB	750 GB

Table 1: Load statistics of different InterMine powered databases. The build time includes creating and storing objects, post-processing and making backups.

InterMine is the system behind a number of data mining warehouses, including:

- FlyMine for *Drosophila* data (http://www.flymine.org/)
- modMine as a data repository for the modENCODE project (http://intermine.modencode.org/)
- YeastMine for budding yeast data (http://yeastmine.yeastgenome.org/)
- RatMine for rat data (http://ratmine.mcw.edu
- metabolicMine for human metabolic diseases (http://www.metabolicmine.org/)

To give an idea of the performance of the InterMine system, an overview of statistics about a sample of currently active Mines maintained by the InterMine group is shown in Table 1. Depending on the machine and data types, around 100,000/objects per minute is currently a typical average load rate.

3 Data import and integration

3.1 The data model

InterMine is data-model agnostic and can operate on any data model but we provide a core data model specifically for handling biological data based on the Sequence Ontology (Eilbeck et al., 2005). We use an SO-based core model for a number of reasons. Firstly, SO is already used by a number of Model Organism Databases (MODs) based on the Generic Model Organism Database (GMOD) framework, including FlyBase, WormBase, SGD, RGD and MGI, among others. Using an SO-based core model facilitates easy integration of data from these MODs, as well as facilitating interoperability. Furthermore, it is an established sequence ontology, with its core set generally undergoing relatively minor changes (mainly additions of new terms). The InterMine data model is extensible and customisable by editing an XML file, so when changes to SO do happen (Mungall et al., 2011), the data model can easily be modified to take them into account. Most recent SO changes have not affected the data model used by InterMine, but in case of major changes, we provide users with update scripts for switching to the updated ontology, while keeping the data intact.

The core biological model has been expanded for individual InterMine instances in order to include, for example, species-specific features or particular types of data that are of interest to the users and are not covered by the SO (e.g. interaction data, or publications details). The ease of doing this, with the rest of the system being automatically updated, is one of the advantages of using InterMine. In these cases, it is the responsibility of the individual databases to ensure correct model migration between versions, if the added elements of their core model change.

3.2 Data import

InterMine instances are built from various data sources; for example, UniProtKB, gene interactions, and GO (gene ontology) annotations each have their own data format. To facilitate the integration of different data sources, data parsers written in Java are provided. The use of data represented by a particular standard facilitates the incorporation of future data into the database. For example, interaction data can be represented by the PSI-MI standard and by supporting this standard in InterMine we can easily accommodate future data published in this format. The details of the data integration modules that InterMine comes equipped with are shown in Table 2. Parsers have been developed in response to demand from the InterMine user community, and we will continue to respond to such demands in the future.

Table 2: Data converters used as part of InterMine.

Source	Data	Data types loaded
arrayexpress-atlas	Loads ArrayExpress .json files retrieved from EBI web service.	genes, expressionItem
biogrid	Loads genetic and protein interaction data from BioGRID. These data include both high-throughput studies and conventional focused studies and have been curated from the literature.	genes, proteins, interactions
biopax	Loads data from files in BioPAX level 2 format	pathways, proteins, genes
chado-db	Loads data from a Chado database.	Any Chado features, eg. chromosome location, genes, proteins.
ensemb-corel	Load Ensembl data from a downloaded MySQL database or access via script using their API	chromosomes, genes, transcripts, exons, protein sequences, CDSs
ensembl-compara	Load Ensembl compara data from public BioMart	homologues

Table 2 – continued from previous page

Source	Data	Data types loaded
ensembl-snp	Load SNP data from a downloaded Ensembl MySQL database	SNPs, chromosomes
entrez-organism	All other sources refer to organisms only by their NCBI taxonomy id. This source will select the taxonIds loaded into the Organism class, fetch details via the Entrez web service and fill in the organism names in the database.	updates fields for organism created by other sources
fasta	Load features and their sequences. Will create a feature for each entry in a FASTA file and set the sequence, the class of the feature to create is set for the whole file.	feature, sequence
gff	Loads features from files in GFF3 format.	features
go-annotation	Load gene association files that link GO terms to genes or proteins.	genes, Gene Ontology terms
go	Load the Gene Ontology term ids, names and definitions, and the relationships between terms.	Gene Ontology terms
intact	Load interactions data from IntAct.	genes, interactions
intermine-items-xml-file	Use this source to load Items XML conforming to the data model directly into the production database.	any
intermine-items-large-xml-file	Use this source to load Items XML conforming to the data model into the production database, this uses an intermediate database to allow it to cope with very large files that would otherwise cause memory problems.	any
interpro	Loads InterPro	protein domains
kegg-orthologues	Loads homologues from KEGG	homologues, genes
kegg-pathway	Loads pathways from KEGG	pathways, genes
miranda	miRBase Targets from the Sanger Institute	MiRNATargets, MRNAs, genes
pdb	Data from PDB	proteins, protein structures
protein-atlas	Read Human Protein Atlas expression data.	genes, tissues
psi-mi-ontology	Loads the file psi-mi.obo file.	ontology terms
pubmed	data from pubmed	publications
reactome	Loads pathways from Reactome	pathways, genes
so	This source loads no data but adds a class in the data model for terms in the sequence ontology (SO). SO terms represent biological features such as gene, exon, 3' UTR.	sequence features
treefam	Loads homologue data from TreeFam	genes, homologues
uniprot	Loads protein information from UniProtKB XML files.	protein sequences, lengths and molecular weights, links between proteins and genes, features located on proteins, UniProt keywords, references and comments. This source can optionally load InterPro protein domains and GO terms
uniprot-fasta	Loads sequences for proteins loaded in UniProt source	sequences
uniprot-keywords	Load definitions of UniProtKB keywords.	updates uniprot keyword definitions
update-publications	All publications are referred to by PubMed id by other sources. This source should be included at the end of the build. It will query all PubMed ids from the database, fetch details from the Entrez web service and fill in Publication objects.	updates publications loaded by other sources

3.3 Extending the data model

Each source can add classes and fields to extend the data model if required, and each source defines how its own data should be integrated. Construction of an InterMine data warehouse (for example, FlyMine) means configuring which sources should be included and specifying the particular organisms or data files to include. During build time, all the model-based components of the system including the database, the Java classes and the web application are automatically derived from the data model. This allows simple and error-free upgrading of the user interface and API as the data model is adjusted and new types of data are added. This system reduces the development time required to update instances of InterMine, and the fact that the data model is extensible makes it possible to incorporate new data types. It also makes it possible to construct comparable data warehouses for different organisms and datasets, creating the potential for developing, for example, cross-species interoperability. Data can also be loaded from an InterMine XML format, allowing the parsing code to be written in a language other than Java.

3.4 Data integration and quality checking

One of the difficulties of data integration comes from the need for complicated cross-referencing of identifiers from different data sources. The information from different databases needs to be correctly interlinked, and the system also needs to be capable of dealing with changing biological knowledge - for example, incorporating information on new gene models into the database, while still keeping the older datasets usable. InterMine tackles this using an identifier resolution system. In brief, when a data file is loaded, the identifier is checked against an ”ID resolver” - a map connecting the identifier to a collection of synonyms and cross-references. The ID resolver is created using information from the relevant model organism databases.

The process of data integration also relies on user-specified data priorities. In the case of conflicts of values from different sources, the value coming from a higher priority source is considered to be the accurate one. There are no default priorities set - developers setting up an InterMine instance are required to set these for themselves, based on which data sources are considered to be more reliable for a given data type. For example, because of its extensive manual curation, FlyBase (Gelbart et al., 1997) is considered to be the authoritative source of gene information for Drosophila in FlyMine (Lyne et al., 2007), while UniProt (UniProt Consortium et al., 2008) is considered to be the authoritative source of protein information, so the priorities are set accordingly.

The build system allows for the integration of data sets of varying size and complexity. It can be configured to make regular data backups, saving time and effort by having restore points for the database. Post-processing tasks can manipulate the data and add extra information after all data has been imported - for example, gene or chromosome lengths can be calculated. Further to this, a set of both manual and automated data quality checks also exist. Data download scripts check for new updates and data parsers include a number of data validation and integration checks. Quality checks are also run post-build. Consistency checks such as looking at the number of database objects created and checking for duplicate identifiers and empty fields help highlight data issues. The standard post-build quality check protocol runs scripts that execute a collection of both simple and complex queries, which confirm that the data have been integrated correctly and flag any inconsistencies. The scripts also compare the new database build to the previous build (if available), and indicate the percent change between the two. This improves database quality by highlighting any problems that have been introduced during the new database build.

3.5 Data types

A large number of data types have been integrated into the various InterMine instances. These data types range from basic genome annotation and protein information to experiment data such as gene expression and ChIP-seq results. An overview of the types of data included in different InterMine instances is shown in Figure 1.

As described, there are a number of quality checks looking at consistency and technically accurate data integration - e.g. highlighting and resolving missing, duplicate or inconsistent fields. However, InterMine does not automatically check for contradictory facts - differences in findings between the different datasets. InterMine is not a curation tool, and it is up to the database developers working with individual InterMine instances to decide what datasets to include, and how to prioritise them in terms of reliability. This means that, while data integration will be performed correctly, it is up to the developers to choose data in an informed manner, and up to the users to interpret the data depending on its source. All integrated data is displayed, and in the case of contradictory facts, the database users can decide for themselves which source to believe. This means InterMine can be used as a tool for highlighting inconsistencies between different datasets.

4 System architecture

The InterMine system is based around the ObjectStore - a custom object/relational mapping system implemented in Java which has been optimised to support read-only performance. The read-only performance gives speed benefits as extensive indexing can be performed during the database build. The ObjectStore can be accessed from the web application and through web services, and executes queries in a PostgreSQL relational database. Here we give the details of the ObjectStore and explain the process of query optimisation.

4.1 Object/Relational mapping

The ObjectStore is designed for quick load times and high query performance in a read-only database. Although written in Java, the ObjectStore does not share Java's restricted type semantics and can model any directed acyclic graph of types, such as those represented by multiple inheritance. Techniques that are designed to manage the presence of such objects include the de-normalisation of tables and a separation between object storage and field value storage to minimise table reads when querying.

A data model is defined at the object level by an XML file. Java objects, the relational database schema and all model-specific parts of the web application are generated automatically, reducing the maintenance overhead when data model changes are required. Data are loaded as Java objects or as XML conforming to the specified model. Integration of data from multiple sources is configured to define how equivalent objects from different sources should be merged. As different data sources may provide different fields, multiple 'keys' can be defined for a particular type. For example, 'Gene' objects may be merged according to an 'identifier' field or a 'symbol' field. As described above (Section 3.4), a priority configuration system is used to resolve conflicts between data sources.

4.2 Query Optimisation

Before final execution, SQL queries are passed to the QueryOptimiser (a Java program developed to enhance InterMine performance), which is able to re-write any SQL query to make use of precomputed tables (analogous to materialised views). The whole query or parts of it may be replaced by

	FlyMine	modMine	metabolicMine
**Genome Annotation**			
sequence	✗	✗	✗
genes	✗	✗	✗
transcripts	✗	✗	✗
exons	✗	✗	✗
regulatory regions	✗	✗	
microarray probes (expression/tiling)	✗	✗	
ESTs	✗	✗	
insertions / deletions	✗		✗
**Proteins**			
sequence	✗	✗	✗
feature locations	✗	✗	✗
domain content	✗	✗	✗
interactions	✗		✗
3D structure	✗		
microarray experiments	✗	✗	✗
in situ hybridisations	✗		
**Genetics**			
alleles	✗		✗
SNPs			✗
interactions	✗	✗	✗
mutant lines			✗
transgenics lines			✗
strains		✗	✗
gene ontology	✗	✗	✗
mammalian phenotype			✗
**Other**			
RNAi screens	✗	✗	
disease	✗		✗
phenotypes	✗	✗	✗
anatomy	✗	✗	
pathways	✗	✗	✗
publications	✗	✗	✗
**Comparative**			
computed orthologues	✗	✗	✗
computed paralogues	✗	✗	

Figure 1: Data types present in different instances of InterMine

one or more precomputed tables; estimates of execution time from the PostgreSQL database are used to decide which query will be fastest.

Precomputed tables can be created while the database system is running in production so performance can be adapted to match actual usage. Within the web application, users with the appropriate permissions, super-users (typically developers), can immediately precompute any new template queries to ensure they run quickly. This approach separates the definition of the data model from performance optimisation, making it possible to tailor the performance of an InterMine warehouse for types of queries not known at design time. A particular benefit of using this system is that the queries can be optimised without the need to de-normalise the data schema. Additionally a cache is used to speed up the performance by using information from previously run queries.

5 Web application

5.1 Features

InterMine comes with a web application that includes flexible query capabilities and a number of analysis and visualisation features. The workflow of the web application is shown in Figure 2. Searches can be run using template queries (saved searches for performing common tasks) or custom written ones made using the QueryBuilder. Query results can be exported, analysed as lists or the report pages of individual objects from the results table query results can be explored. Lists created from query results can in turn be used for running further queries.

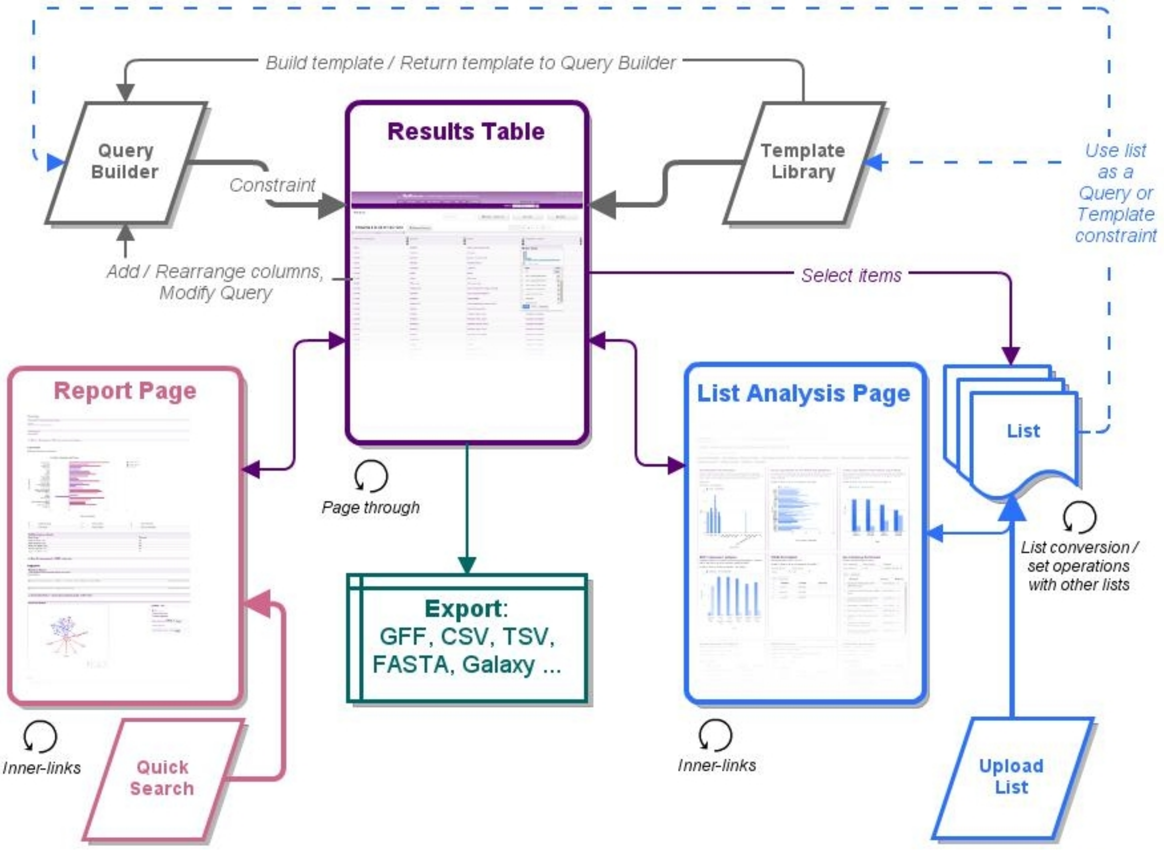

Figure 2: Web application workflow structure

Keyword search (Figure 3) enables users to search across the database, quickly finding, for example,

genes or experiment datasets of interest. Logic operations are available, and faceted filtering can be used to narrow down the selection to the data of interest.

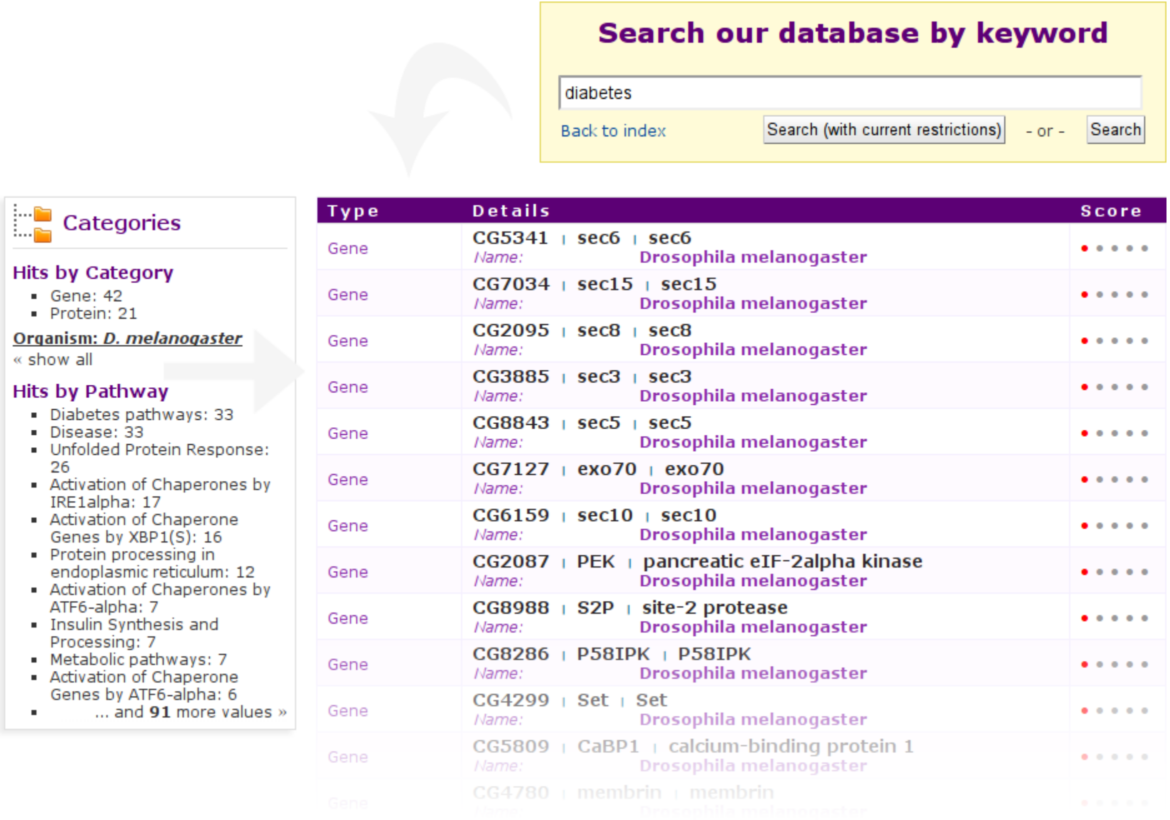

Figure 3: Keyword search for the word 'diabetes' retrieves a range of results associated with the disease, including genes, proteins and pathways. The faceted filtering on the left allows the user to further narrow down the results set.

The search results are linked to report pages (Figure 4), which display a rich collection of information. The data presented on the report page is hyperlinked both to the corresponding database objects and to external websites, and can be explored and queried further. For example, someone starting from a gene search can click on a pathway that the gene is involved in, save a list of all the genes associated with that pathway, and use that for further searches.

List analysis pages contain analysis 'widgets' (Figure 5) - tools showing summary graphs or statistics, such as the statistically enriched GO terms and publications for a given list of genes. Information about things such as known interactions and pathways are also displayed. The list analysis pages are a very popular feature of InterMine because they provide a useful summary of information from a range of integrated data. Because all data objects have report pages and all query results can be converted to lists, this enables a natural exploration of different aspects of, for example, gene lists of interest, by browsing through related pathways and processes. This is helpful both for an exploratory analysis, where the aim is to expand the connections from a particular topic of interest, and for a funneling workflow, where the aim is to reduce a large starting list to a small number of promising candidates.

5.2 User authentication and workspace

Logging in is not required for using InterMine - for users who are not logged in, details of lists are saved for the duration of the session using cookies. However, logging into an InterMine instance

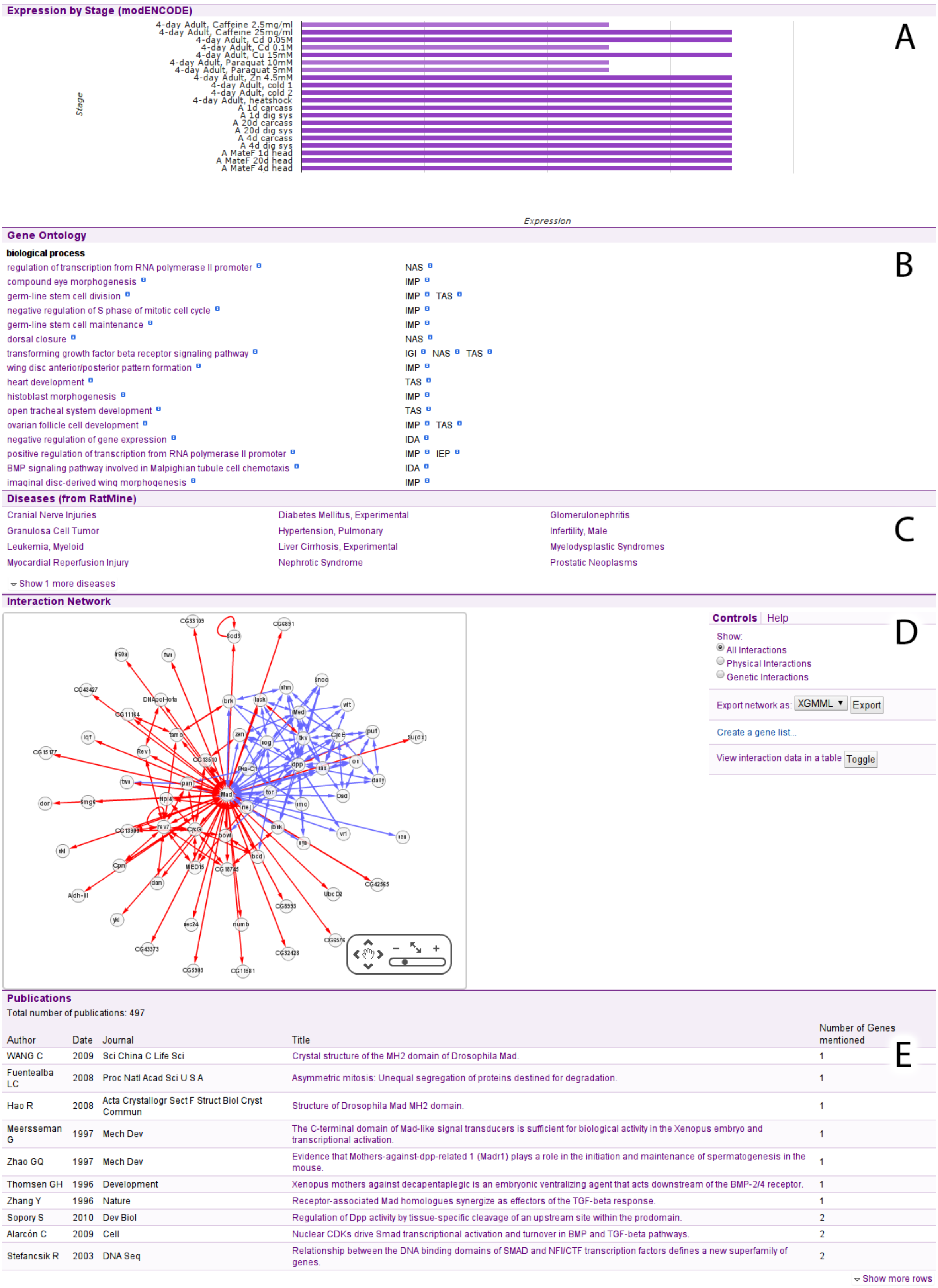

Figure 4: The InterMine report pages show a collated view of the information about a given object. Here we show a small number of features of a gene report page, showing information on gene expression (A), gene ontology (B), known diseases (C), interactions (D), and associated publications (E).

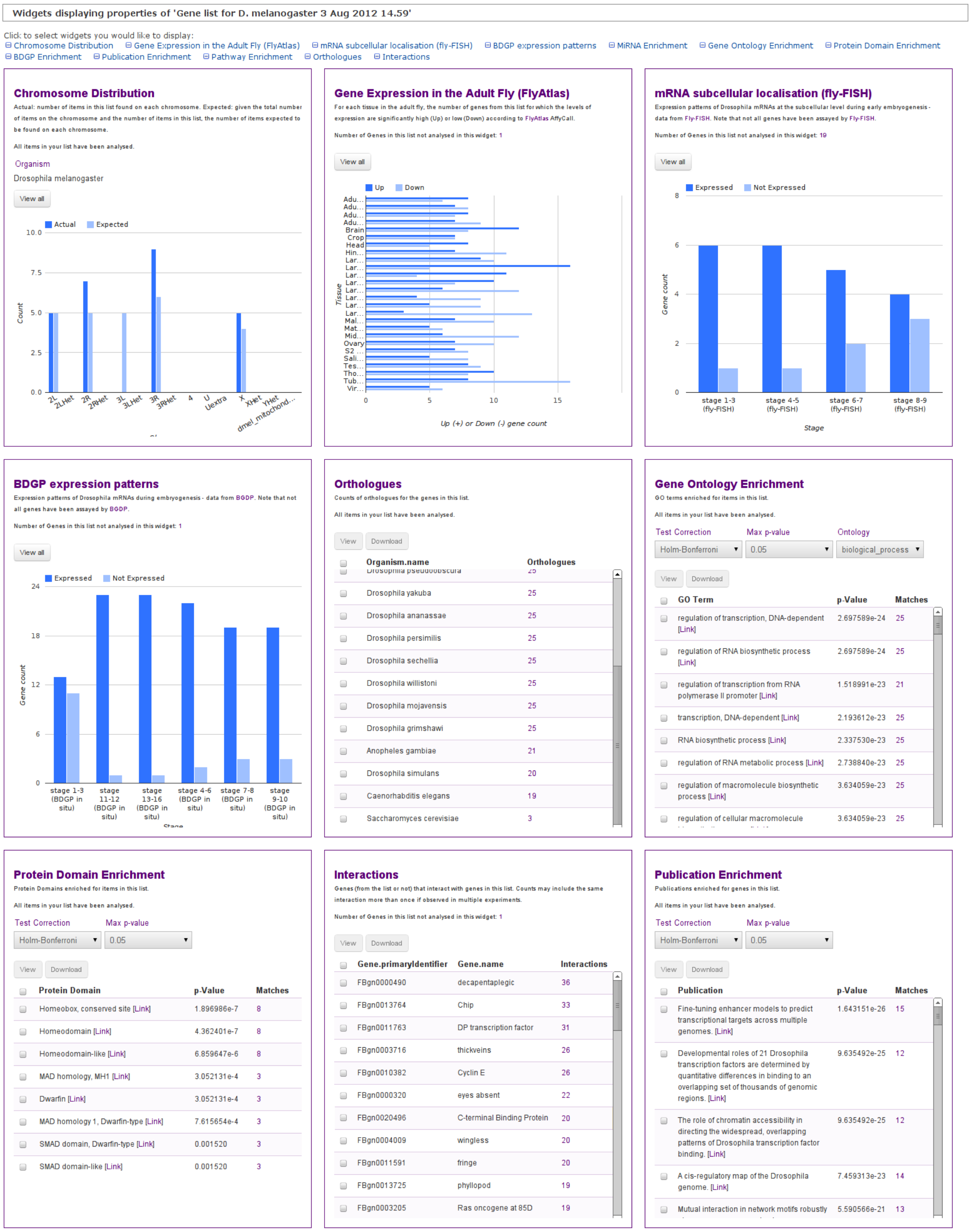

Figure 5: The widgets included as part of a list analysis page perform a number of analyses. Shown here are the FlyMine widgets for exploring chromosome distribution, gene expression patterns (including both adult data from FlyAtlas and embryo data from BDGP), mRNA subcellular localisation, interactions, and also for the enrichment of Gene Ontology terms, protein domains, and publications. A list analysis results page that includes these is automatically generated every time the user creates a new list, facilitating rapid exploration and serendipitous discovery.

does have advantages as it saves the lists and queries permanently under a given username in a user's private 'MyMine' workspace. One way of logging in is by registering as a user of a given InterMine instance. It is also possible to log in by authenticating with OpenID providers such as myId or Yahoo. A user's lists are only saved in the single InterMine instance that they were created in - e.g. logging into YeastMine with OpenID will not automatically transfer their gene lists from RatMine. Lists can, however, be exported from one InterMine instance to another. User input descriptions (where applied) are also saved alongside the lists.

Different classes of user have different capabilities: super-users can configure the positioning of content on report pages and the front page using a web-based tagging system. In addition they can use the web application to precompute and publish template searches, as well as creating and useful public lists, e.g. lists of specific manually curated genes.

5.3 Query capabilities

As described, InterMine has been designed to enable the efficient construction and flexible querying of large databases with complex data integration requirements. Any parts of the data model can be queried, and InterMine also includes functionality for querying features which overlap specific genome ranges. Specific distances upstream and downstream of genes can also be represented to enable querying for genes that are near other features.

Template queries can be created for commonly run searches and there is a sophisticated QueryBuilder (Figure 6) for constructing advanced custom queries. Because both the templates and custom queries can be run within the web application using a graphical interface, querying the data does not require programming knowledge. Queries can also be exported and imported as XML and thus can be shared between users as well as between InterMine instances. Query results can be saved and exported from the InterMine web application in a range of common formats, for example as tab-delimited, comma-delimited, GFF3 or BED files and the users can customise the data fields to export. Data can also be exported directly into Galaxy.

5.4 Embedding external tools

External display tools such as GBrowse (http://gmod.org/wiki/GBrowse), JBrowse (http://jbrowse.org/) and Cytoscape Web (http://cytoscapeweb.cytoscape.org/) have been integrated within the web application and it is straightforward to add others. In the case of GBrowse, data are not served directly from the features stored in InterMine, but from a GBrowse-specific database. This database is loaded as part of the InterMine build process, so ensuring that the same features are stored both in GBrowse and in the InterMine database.

6 Web services

An InterMine web application presents visitors with both a graphical user interface and access to machine readable web services. The web services, to be described in more detail in a forthcoming paper, expose the majority of the functionality underlying an InterMine application through standard HTTP 1.0 method calls (often termed a RESTful web service). This design allows for the greatest variety of clients to be supported directly, in particular it allows for a rich JavaScript client to access InterMine functionality from third party web sites. To support bioinformaticians, InterMine has published libraries to facilitate access to the InterMine API in Java, Perl, Python, Ruby and JavaScript.

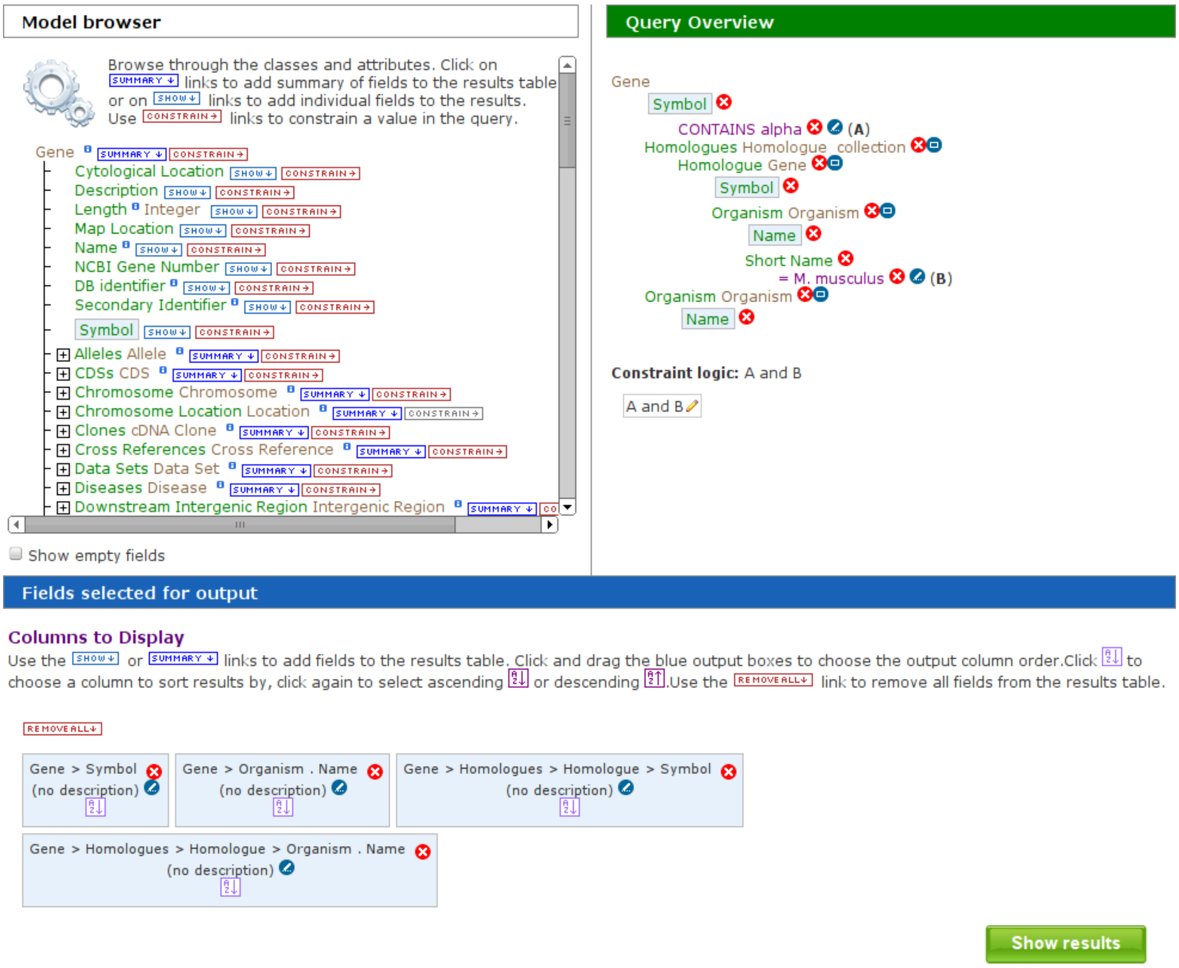

Figure 6: The QueryBuilder provides a graphical interface for browsing through the data model and constructing and editing queries. In this example, the constructed query is searching for all genes containing the string ”alpha” in *Drosophila*, and then also displaying their homologues from the species *M. musculus*.

7 Pros and cons - why use InterMine

With a variety of data solutions available, it can be difficult to choose the most appropriate one for your specific data management requirements. Here we present an overview of the existing solutions, the specific strengths of InterMine, and the situations in which it is an appropriate choice of data platform. There is a lot of overlap between the features available from a number of different data management solutions - however, it is the specific combination of features available together that makes them appropriate for particular uses. While a range of database management systems offer, variously, query flexibility, speed, and the ability to host large quantities of data, an ideal combination of these is rare, with for example, speed often coming at the price of reduced flexibility.

The useful features of InterMine include:

- Facilities for complex data integration. The data model is flexible and extensible, and a range of data parsers are provided to facilitate the data loading. Furthermore, the query optimisation method is constructed around the use of precomputed tables, meaning that the data schema does not need to be denormalized in order to speed up query time.
- Fast, flexible querying. The sophisticated query optimisation means that users can construct and perform a wide range of queries across the data model, while retaining good query speed. The system is also fast enough to deal with large quantities of data - as shown in Table 1, the modMine database contains 130 million objects, and its size with precomputed tables is 750 GB, with metabolicMine being even larger, containing 260 million objects and almost 2000GB including precomputed tables.
- Pre-constructed web interface and analysis widgets. The web application is included with the InterMine package, and is an accessible starting point for first time users. It contains a number of features focused around list analysis (a common need in biology), as well as report pages, template queries and a regions search tool. This setup makes it possible to browse and explore data without any programming knowledge.
- Developed set of APIs and web tools. InterMine can be accessed programmatically, and we provide client libraries for five commonly used programming languages (Python, Perl, Ruby, Java, JavaScript). This enables bioinformatician users to access InterMine functionality without using the web application and to query data from a number of different InterMine instances using a single script, or as part of an automated workflow.
- Highly developed and extensible system. InterMine has been in development for 10 years, and during this time, based on user demand, we have introduced a large number of features. These range from faceted filtering options and enabling Boolean logic and set operations, to table sorting and filtering, a range of standardised export options, integration of other tools such as Cytoscape, and enabling embedding of individual analysis tools as part of external websites. With funding secured for a further 5 years, we plan to continue adding features to InterMine. Furthermore, the open source, extensible framework means InterMine is also open to other developers to build upon.

The above features also come with a set of trade-offs:

- Initial InterMine configuration requires expertise. Although instructions are provided online, setting up InterMine requires some familiarity with command-line tools. It also has dependencies on other open source packages such as Tomcat and PostgreSQL. Establishing the correct configuration of software, if none of it has been set up at the start, can be time consuming.
- Data needs to be loaded into new InterMine instances. Data loaders are provided, but may need to be adapted if data source incompatibilities are found. This is often the case if the data suppliers change formats.

- A new build is required for updates. Having a read-only database provides significant advantages for query performance, and preserves the data in a quality checked, usable form, but requires a time investment for each new build.
- The data model can be confusing to new database users. The large, flexible data model does mean that the model can end up being complex, and possibly confusing to new users. We attempt to bypass this problem by having a range of template searches for common tasks. Furthermore, the user can modify these template searches using the QueryBuilder, thus helping them learn the system.
- The analysis tool speed varies. Recently developed tools, such as the regions tool, are not always suitable for analysis of high throughput data, and deal better with a small number of regions. This is not the case across the board - most of our tools are highly optimised, and adept at dealing with large quantities of input data.

Some of the cons are unavoidable trade-offs, such as the build time requirement for a read-only database. Other ones are issues that we are working to improve, such as setting up a virtual machine on Amazon with an InterMine instance, giving users the ability to use InterMine without having to install and configure third party software such as PostgreSQL and TomCat. In general, however, we think that as a large, established data warehouse system, InterMine has distinct advantages to offer to potential users.

7.1 Comparison to existing systems

While different data integration solutions have their strengths and weaknesses, an ideal data management solution has not yet been found for biological sciences. Instead, a number of established systems exist, and their use is appropriate in different scenarios. While a formal benchmarking review at present does not exist, a basic comparison in a neutral review has been published: Triplet and Butler (2011) looked at effective strategies for data integration, and in the process compared InterMine to four other systems - BioMart, BioXRT, Open Genome Resource (OGeR) and PROFESS. We show quotations from Triplet and Butler (2011) in Table 3, in order to give an idea of the way InterMine compares to other available general data warehousing frameworks.

InterMine is a robust, established system suitable for large data warehouses containing complex data, intended to be maintained as a resource, and used for multiple analyses. Users have access to an established data warehouse framework with advanced features, and the web application included means that the resource can easily be released for public use. There is a time investment that goes into setup and maintenance, so setting up an InterMine instance is less appropriate for users looking for a quick solution or simple analysis of small scale data. However, InterMine implementation through virtual instances will go some way to mitigating set-up costs, and this work is in progress.

InterMine doesn't provide facilities for federation, but otherwise matches the features of the other available data warehouses (an API with libraries in 5 languages was introduced for InterMine since the Triplet and Butler (2011) publication). In addition, InterMine has several unique features, including a range of data parsers, flexible query facilities allowing access to data at a range of levels, and tracking of data provenance. In general, federation as an approach has the starting advantage that the user can set up a simpler database and be connected to the data from federation partners without having to load the data from each one. This is not the case for InterMine - while some interoperability links exist between different InterMine instances such as the ability to export lists of gene identifiers via orthologues, in general, data has to be loaded separately into each InterMine instance.

The complexity of bioinformatics data introduces a number of issues for data integration (Goble and Stevens, 2008), to which, at present, there is no one solution. One of the contributions that will

System	Advantages	Disadvantages
BioMart	-Tools for federating a variety of biological databases<br>-Unified web-based user-friendly interface for data mining<br>-Supports programmatic access (Perl API, RESTful web services)<br>-Queries defined as a set of successive filters	-Queries limited to only two datasets at once<br>-Not possible to edit or create new filters
BioXRT	-Flexible and extensible database structure<br>-Tools for importing spreadsheets	-All pieces of data are defined as strings of characters<br>-Queries are constrained to string matching - no advanced data mining tools
InterMine	-Parsers for integrating data from numerous formats<br>-Web access to integrated data at a number of levels, from simple browsing to complex queries<br>-Facilities for adding one's own data<br>-User friendly web interface that can be easily customised<br>-Data provenance is tracked	-No tools for classifying or clustering data<br>-Queries don't include similarity functions to address annotation errors
OGeR	-Genome sequences and annotations can be automatically downloaded<br>-Features cross-references to external databases	-No tools for clustering and statistical analysis<br>-No advanced data mining tools beyond sequence allignment
PROFESS	-Unified text field for mining data from any integrated database<br>-Provides clustering and aggregation tools for statistical analysis of large datasets<br>-Features a user friendly modular web interface	-Query system is based on a non-customisable set of filters<br>- Does not support similarity functions between standard BLAST searches

Table 3: Quotations from a review of strategies for effective data integration by Triplet and Butler (2011), describing the advantages and disadvantages of different data warehouse systems.

pave the way towards better bioinformatics data management and analysis is the implementation of community-wide standards, such as exemplified by the development of a range of ontologies including Gene Ontology Ashburner et al. (2000). Semantic Web technology utilizes ontologies, and shows a lot of promise as a solution for data integration on a large scale that is not limited to individual software systems (Chen et al., 2012; Antezana et al., 2009). This technology may well emerge as a good solution to the problem of biological data integration, yet at least some of it is still a research problem in its own right. Since it is built on a set of generic standards (e.g. JSON), InterMine is in a good position to expand its framework to exploit the wider adoption of biological Semantic Web principles, or indeed any other emerging data integration solutions. In conclusion, the InterMine system presents an established solution for the setting up of extensive resources for integrating large and complex biological datasets.

References

Antezana, E., Kuiper, M. and Mironov, V. (2009). Biological knowledge management: the emerging role of the semantic web technologies, *Briefings in bioinformatics* **10**(4): 392–407.

Ashburner, M., Ball, C., Blake, J., Botstein, D., Butler, H., Cherry, J., Davis, A., Dolinski, K., Dwight, S., Eppig, J. et al. (2000). Gene ontology: tool for the unification of biology, *Nature genetics* **25**(1): 25.

Chen, H., Yu, T. and Chen, J. (2012). Semantic web meets integrative biology: a survey, *Briefings in Bioinformatics* .

Eilbeck, K., Lewis, S., Mungall, C., Yandell, M., Stein, L., Durbin, R. and Ashburner, M. (2005). The Sequence Ontology: a tool for the unification of genome annotations, *Genome biology* **6**(5): R44.

Gelbart, W., Crosby, M., Matthews, B., Rindone, W., Chillemi, J., Twombly, S., Emmert, D., Ashburner, M., Drysdale, R., Whitfield, E. et al. (1997). FlyBase: a *Drosophila* database. The FlyBase consortium., *Nucleic Acids Research* **25**(1): 63.

Goble, C. and Stevens, R. (2008). State of the nation in data integration for bioinformatics, *Journal of biomedical informatics* **41**(5): 687–693.

Lyne, R., Smith, R., Rutherford, K., Wakeling, M., Varley, A., Guillier, F., Janssens, H., Ji, W., Mclaren, P., North, P. et al. (2007). FlyMine: an integrated database for *Drosophila* and *Anopheles* genomics, *Genome Biology* **8**(7): R129.

Mungall, C., Batchelor, C. and Eilbeck, K. (2011). Evolution of the sequence ontology terms and relationships, *Journal of Biomedical Informatics* **44**(1): 87–93.

Triplet, T. and Butler, G. (2011). Systems biology warehousing: Challenges and strategies toward effective data integration, *DBKDA 2011, The Third International Conference on Advances in Databases, Knowledge, and Data Applications*, pp. 34–40.

UniProt Consortium et al. (2008). The universal protein resource (UniProt), *Nucleic Acids Res* **36**(D190-D195): 61–79.
